# Hyperhidrosis: Anatomy, Pathophysiology and Treatment with Emphasis on the Role of Botulinum Toxins

**DOI:** 10.3390/toxins5040821

**Published:** 2013-04-23

**Authors:** Amanda-Amrita D. Lakraj, Narges Moghimi, Bahman Jabbari

**Affiliations:** 1 Department of Neurology, Yale University School of Medicine; New Haven, CT 06520, USA; E-Mail: bahaman.jabbari@yale.edu; 2 Department of Neurology, Case Western Reserve University; Cleveland, OH 44106, USA; E-Mail: narges_moghimi@yahoo.com

**Keywords:** botulinum neurotoxins, hyperhidrosis, iontophoresis, double blind, axillary, palmar, gustatory, compensatory, topical agents, oral agents

## Abstract

Clinical features, anatomy and physiology of hyperhidrosis are presented with a review of the world literature on treatment. Level of drug efficacy is defined according to the guidelines of the American Academy of Neurology. Topical agents (glycopyrrolate and methylsulfate) are evidence level B (probably effective). Oral agents (oxybutynin and methantheline bromide) are also level B. In a total of 831 patients, 1 class I and 2 class II blinded studies showed level B efficacy of OnabotulinumtoxinA (A/Ona), while 1 class I and 1 class II study also demonstrated level B efficacy of AbobotulinumtoxinA (A/Abo) in axillary hyperhidrosis (AH), collectively depicting Level A evidence (established) for botulinumtoxinA (BoNT-A). In a comparator study, A/Ona and A/Inco toxins demonstrated comparable efficacy in AH. For IncobotulinumtoxinA (A/Inco) no placebo controlled studies exist; thus, efficacy is Level C (possibly effective) based solely on the aforementioned class II comparator study. For RimabotulinumtoxinB (B/Rima), one class III study has suggested Level U efficacy (insufficient data). In palmar hyperhidrosis (PH), there are 3 class II studies for A/Ona and 2 for A/Abo (individually and collectively level B for BoNT-A) and no blinded study for A/Inco (level U). For B/Rima the level of evidence is C (possibly effective) based on 1 class II study. Botulinum toxins (BoNT) provide a long lasting effect of 3–9 months after one injection session. Studies on BoNT-A iontophoresis are emerging (2 class II studies; level B); however, data on duration and frequency of application is inconsistent.

## 1. Introduction—Definition and Incidence

Hyperhidrosis (excessive sweating) is a chronic autonomic disorder that can be debilitating leading to emotional and social embarrassment, as well as occupational, physical and psychological disability [1]. In a majority of cases, the cause of hyperhidrosis is unknown [2]. Primary hyperhidrosis starts in childhood and affects 0.6%–1% of the population [3]. A familial variant with autosomal dominant inheritance is now recognized with some families linked to an abnormality of chromosome 14q [4]. The diagnostic criteria for hyperhidrosis includes excessive sweating that lasts at least six months without any obvious cause and has at least two of the following features: impairs daily activities, a bilateral and relatively symmetric pattern of sweating occurring at least once per week, an age of onset younger than 25, cessation of focal sweating during sleep, or positive family history [5]. Secondary hyperhidrosis can be drug-induced (for example with sertraline), toxin-induced (acrylamide) [6], caused by a systemic illness (endocrine and metabolic disorders, neoplasms, spinal cord lesions), by congenital disorders such as familial dysautonomia (Riley-Day syndrome) or it can be compensatory [2]. Compensatory hyperhidrosis is a phenomenon in which there is increased sweating in parts of the body unrelated to the location of treatment or in the case of surgery, unrelated to surgery or anatomy [7]. It is often seen in segments below the level of sympathectomy, which is performed for treatment. Gustatory hyperhidrosis (usually involving the face) can be familial or occur in association with trauma or other local insults. One epidemiologic survey in 2004 estimated that as many as 0.5% of the US population may be suffering from the debilitating effects of hyperhidrosis with major interference in daily activities [8]. 

## 2. Objective

The objective of this paper is to provide a comprehensive review of hyperhidrosis providing information on anatomy, physiology, and current treatment methods with an emphasis on the role of botulinum neurotoxins (BoNTs). The evidence of efficacy is presented according to the guidelines of the American Academy of Neurology [9]. 

## 3. Method

Information for this paper was collected by searching the Yale Medical Library Database that utilizes a wide range of scholarly search engines including but not limited to PubMed, Erasmus, Ovid, EBSCO and Cochrane databases. Literature was searched from the timeline 1960 to present, including literature ahead of print and articles not in English. Terms used for search included “hyperhidrosis”, “sweating”, “double blind”, “therapy”, “treatment”, “axillary”, “palmar”, “gustatory”, “facial”, “botulinum toxin”, “botulinum neurotoxin”, “oral”, “topical” and “surgical” in various combinations. 

## 4. Anatomy

Hyperhidrosis occurs as a primary process of autonomic neuronal dysfunction. This dysfunction tends to occur in areas where there is a higher concentration of eccrine glands such as the palms, soles, and axillae, which are sweat-producing glands [10]. Less common sites are the scalp or face [11]. The nerves that innervate sweat glands are sympathetic, postganglionic and have acetylcholine as their primary neurotransmitter [12]. These fibers consist of unmyelinated class C fibers [7]. Norepinephrine and vasoactive intestinal peptide (VIP) may play a role, but neither of these amplifies cholinergic sweat secretion [13].

A central sudomotor efferent pathway is suggested for hyperhidrosis with the following connections: (1) cerebral cortex to hypothalamus; (2) hypothalamus to medulla; (3) fibers crossing in the medulla oblongtata and travelling to the lateral horn of the spinal cord; (4) the lateral horn to sympathetic ganglia; and (5) sympathetic ganglia to sweat glands as postganglionic C fibers [7]. Because the sympathetic fibers arising from the hypothalamus cross mostly at the level of the pons, and most of this crossing is completed in the medulla oblongate, lesions in the medulla may cause altered sweating, such as the ipsilateral anhidrosis seen in Horner’s syndrome. 

## 5. Physiology

Sweat glands on the palms and soles alone are activated mostly by emotional stimuli. Frontal and pre-motor projections to hypothalamus probably promote sweating during enhanced emotions [14]. It is believed the hypothalamic sweat center, which is in charge of the palms, soles, and in some individuals the axilla, is distinct from the other hypothalamic sweat centers and is actually under exclusive control of the cortex, with no input from the thermosensistive elements. Because emotional sweating does not occur during sleep or sedation, one of the criteria for primary hyperhidrosis is that the individual does not experience sweating during sleep. Sympathetic cholinergic nerves activate both thermoregulatory and emotional sweating and are controlled by different central nervous system neurons. It is possible that Primary hyperhidrosis is due to abnormal central control of emotional sweating given that it affects the same body areas as those affected in emotional sweating (hands, feet, and axillae) [15].

## 6. Treatments

Pharmacological treatments of hyperhidrosis include topical, oral and iontophoretic treatments as well as BoNT injections. To date an established level of evidence (Level A, two or more Class I papers) [9] (Table 1) exists only for BoNT-A treatment of axillary hyperhidrosis. 

**Table 1:** AAN classification of evidence [9].

Class	Criteria	Level of evidence	Recommendation
I	Prospective, randomized, controlled, outcome masked, representative population with criteria A–E *	A: Two or more Class I studies	Established as effective, ineffective, or harmful
II	Prospective, matched cohort, representative population, masked outcome and meets A–E * OR RCT with one criteria in A–E * lacking	B: At least one Class I or two Class II	Probably effective, ineffective, or harmful and recommended
III	Controlled trial **, representative population, outcome independent of patient treatment	C: At least one Class II	Possibly effective, ineffective or harmful, may be used at discretion of clinician
IV	Uncontrolled study, case series, case report or expert opinion.	U	Data inadequate or conflicting

* Criteria A-DE: A = Primary outcome(s) clearly defined, B = exclusion/inclusion criteria clearly defined, C = Adequate accounting for drop-outs and cross-over with numbers sufficiently low to have minimal potential for bias, D = relevant baseline characteristics or appropriate statistical adjustment for differences, E = For non-inferiority or equivalence trials claiming to prove efficacy for one or both drugs, the following are also required: 1. The standard treatment used in the study is substantially similar to that used in previous studies establishing efficacy of the standard treatment (e.g., for a drug, the mode of administration, dose, and dosage adjustments are similar to those previously shown to be effective); 2. The inclusion and exclusion criteria for patient selection and the outcomes of patients on the standard treatment are substantially equivalent to those of previous studies establishing efficacy of the standard treatment and 3. The interpretation of the results of the study is based on an observed-case analysis. ** Including well-defined natural history controls or patients serving as their own controls.

### 6.1. Topical Agents

Aluminum salts are the main topical agents for hyperhidrosis. Their mechanism of action is attributed to either an interaction between aluminum chloride and keratin in the sweat ducts (duct closure) or to a direct action on the excretory eccrine gland epithelium [16]. They are only effective in milder cases of hyperhidrosis, and duration of effect is often limited to 48 h [1]. The most common side effect is skin irritation, likely related to high salt concentration [17].

Topical agents have been studied for use in all forms of hyperhidrosis (axillary, palmoplantar, and gustatory). Although more commonly used for axillary and palmar hyperhidrosis, double blind studies available in the literature and presented in this paper focus on gustatory hyperhidrosis. Of four double blind studies published in the literature on these agents, two are class II and two are class III, rendering a B level of evidence for these agents (two class II, probably effective) (Table 2). 

**Table 2:** Double blind placebo controlled studies of topical and oral agents in hyperhidrosis.

Agent	Author and year	Type of hyperhidrosis	*N*	Study design	Class	Findings	Side effects
Topical Aluminium Chloride Hexahydrate 25% in Ethanol	Glent-Madsen *et al*., 1988	AH	30	Randomized, double-blind, half-sided experiment	III	25% aluminum chloride in ethanol alone was effective in all pts	Skin irritation
Topical Glycopyrrolate	Shaw *et al*., 1997	Gustatory (Frey’s syndrome)	13	Double-blind, PBO-controlled, crossover study	II	All pts experienced significant improvement. Glycopyrrolate reduced the sweat response to a challenge by 82% (*p* < 0.01). The frequency of episodes of gustatory sweating also reduced by 51% (*p* < 0.01), with a nearly 100% reduction in the frequency of severe sweating (*p* < 0.01)	Eczematous reaction in one patient
Topical Glycopyrrolate	Hays 1978	Gustatory (Frey’s syndrome)	16	Double blind clinical trial	III	Topical glycopyrrolate(0.5% and 1.0% ) abolished gustatory sweating for several days after single application.	No significant side effects
Topical 2% Diphemanil Methylsulfate (Prantal)	Laccourreye *et al*., 1990	Gustatory (Frey’s syndrome)	15	Double blind study	II	Partial relief in 33.3% and total relief in 40%. Duration of relief varied from 2 to 4 days.	Dryness of the mouth noted in two pts.
Oral Menthatheline Bromide (Vagantin)(systemic anticholinergic)	Hund *et al*., 2004	AH and PH	41	Randomized, PBO-controlled, double blind clinical trial	II	Mean axillary sweat production decreased in the treated arm from 89.2 ± 73.4 mg/min prior to therapy to 53.3 ± 48.7 mg/min during therapy (*p* = 0.02). No change in palmar sweat.	Dry mouth
Oral Menthatheline Bromide (Vagantin)(systemic anticholinergic)	Muller *et al*., 2012	PH, AH or Plamo-Axillary	339	Multicenter, randomized, PBO controlled trial, blinding not accurately described	II	50mg three times a day: improved DLQI, HDSS, and decreased mean axillary sweat production (*p* = 0.004).	Dry mouth frequently reported
Oral Oxybutynin	Ghaleiha *et al*., 2012	Hyperhidrosis secondary to Sertaline	140	double-blind, PBO-controlled	I	Improved HDSS in the drug compared to PBO group, *p* ≤ 0.05	GI and GU symptoms, sedation, dry mouth,
Oral Oxybutynin at low doses	Nelson *et al*., 2012	PH, AH, and plantar	50	Prospective, randomized, single blinded(patient blinded), PBO controlled	II	5mg twice daily caused moderate to marked improvement in PH or AH, (70%) *versus* 27.3% in PBO (*p* < 0.001). Moderate or great improvement in plantar hyperhidrosis (>90%) compared to 13.4% in PBO (*p* < 0.01)	Dry mouth (frequent) in 47.8%

PH: Palmar Hyperhidrosis; AH: Axillary Hyperhidrosis; PBO: placebo; pts: patients; DLQI: Dermatology Life Quality Index; HDSS: Hyperhidrosis Disease Severity Score.

### 6.2. Oral Agents

Anticholinergic agents (glycopyrrolate, menthatheline bromide, oxybutynin) and alpha-adrenergic agonists (clonidine) are most commonly used in clinical practice. Anticholinergic agents work by competitive inhibition of acetylcholine at muscarinic receptors (affinity for M3 receptors in glandular issue). Optimum doses for each of these agents are still under study [18], however the following dosages are often clinically practiced: glycopyrrolate 1–2 mg twice a day [19], oxybutynin 5–7.5 mg twice a day [20], and methantheline bromide 50 mg twice a day [21]. Side effects can be very disabling and include dry mouth, blurring of vision, urinary hesitancy, dizziness, tachycardia, and confusion [19]. Contraindications include: myasthenia gravis, pyloric stenosis, narrow angle glaucoma and paralytic illeus. Also, caution should be used in patients having gastroesophogeal reflux disease, glaucoma, bladder outflow obstruction, and cardiac insufficiency. Clonidine, given as 0.1mg twice a day, is an antihypertensive agent that by enhancing the function of alpha adrenergic receptors (α2 agonist) inhibits the sympathetic output. Side effects include dry mouth, dizziness, constipation, sedation and symptomatic decrease in blood pressure [19]. 

Oral agents have been implicated for use in all subtypes of hyperhidrosis (axillary, palmoplantar, craniofacial/gustatory, and generalized). Double blind studies however, are available only for axillary and palmoplantar hyperhidoris. The evidence to date indicates that both oxybutinin (one class I study and one class II study) and menthatheline bromide (two class II studies) are probably effective with Level B evidence individually (Table 2). The efficacy of oral glycopyrrolate and clonidine is suggested only in retrospective studies. Collectively, oral agents have level B evidence (probably effective) with one class I study and three class II studies present in the literature.

### 6.3. Iontophoresis

Iontophoresis is defined as the introduction of an ionized substance through application of a direct current on intact skin [22]. Though the exact mechanism of action is unknown, this technique facilitates transdermal movement of solute ions by generation of an electrical potential gradient. Penetration of neutral compounds is also facilitated. Tap water, anticholinergic agents (glycopyrrolate) and BoNTs are candidates for use in iontophoresis. The latter are less often used due to their large molecular size, which poses a challenge. Tap water iontophoresis must be performed initially every two-three days until therapeutic effect is achieved. Once therapeutic effect is achieved for two weeks, treatment can be done once every two-three weeks [23]. Duration of effect is only a few days with tap water and anticholinergic iontophoresis [19], however iontophoresis with BoNT may provide relief for three months [24]. Out of 27 patients who underwent iontophoresis with BoNT, 100% were found to have statistically significant improvement in sweating via gravimetry (*p* < 0.05) [24,25,26]. The short- lived effect of current methods of electrophoresis makes it undesirable [27]. Minor pain and skin irritation (burning, tingling, erythema and discomfort) may occur and vesicles may also develop [28]. Presence of metallic implants such as cardiac pacemakers, artificial orthopedic joints or bone implants and pregnancy are contraindications [29]. Blinded studies are limited. 

### 6.4. Botulinum Toxin—Studies and Methodology

**Figure 1:**
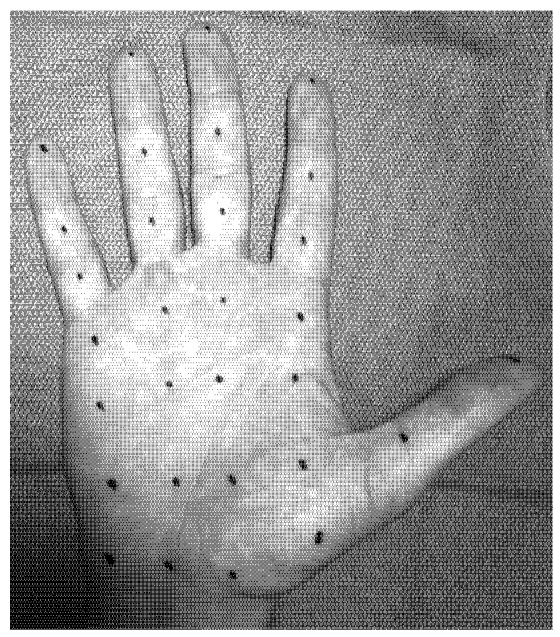
Grid pattern used for palmar injections [32].

**Figure 2:**
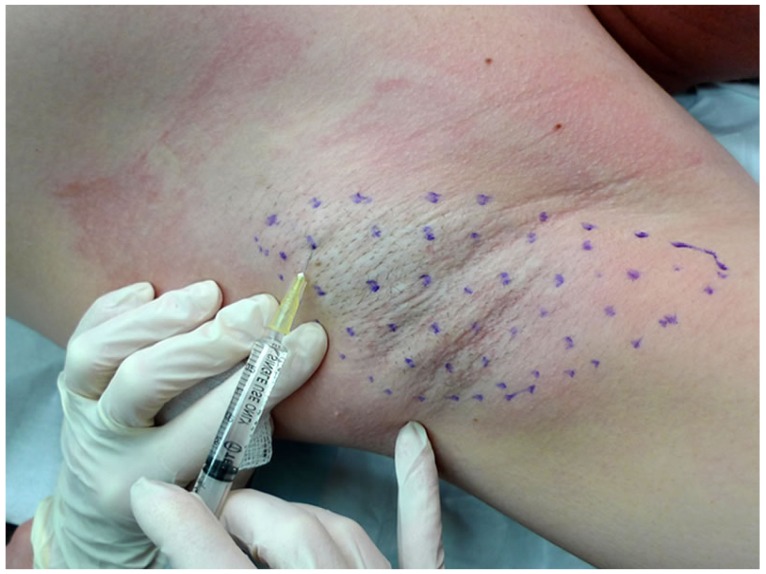
Pattern followed for axillary injections [33].

BoNTs block the release of acetylcholine and a number of other neurotransmitters from presynaptic vesicles by deactivating SNARE proteins. Four types of BoNTs are approved by FDA for clinical use in the USA: onabotulinumtoxinA (A/Ona, Botox), incobotulinumtoxinA (A/Inco, Xeomin), abobotulinumtoxinA (A/Abo, Dysport) and rimabotulinumtoxinB (B/Rima, Myobloc). These toxins use different presynaptic proteins for their site of action. For instance, for A/Abo the protein is Synaptin 25. For B/Rima it is synaptobrevin, also known as vesicle-associated membrane protein (VAMP). The sweat reducing effect of BoNT-A was first observed in asymptomatic volunteers [30]. Intradermal injections are usually carried out in a grid pattern with a small needle (gauge 30) to the depth of few millimeters, with 2–2.5 units of toxin administered at each site (Figure 1, Figure 2). To decrease pain, skin is usually numbed with Emla cream an hour before injection, by application of local anesthetic spray, or both. Absolute contraindications for injection with BoNT include skin infections and allergies to any of the ingredients in BoNT formulations. Relative contraindications include illnesses resulting in muscle weakness (ALS, Lou Gehrig’s), dysphagia (Myasthenia Gravis or Lambert Eaton Syndrome) or respiratory compromise [31]. 

## 7. Axillary Hyperhidrosis (AH)

Axillary Hyperhidrosis (AH) is excessive sweating specifically in the area of the axillae and is usually bilateral in pattern. While it can be continuous, it is more commonly phasic. It may be precipitated by heat or mental stress [34] and is associated with dermatologic complications including pompholyx and contact dermatitis [35]. 

Ten blinded studies (nine with BoNT-A and one with BoNT-B) are reported for use in axillary hyperhidrosis. Six studies compared a BoNT with placebo (Table 3). Two studies compared one BoNT mixed with saline to one BoNT mixed with an anesthetic agent. One study compared the efficacy and side effects of two toxins against each other. Finally, one study compared the efficacy of mode of delivery (*i.e*., injection *versus* iontophoresis). 

**Table 3:** Double blind axillary hyperhidrosis studies comparing toxin *vs.* placebo.

Author & year	*N*	Class	Agent	Dose	Primary outcome	Result	Side effects
Schnider *et al*., 1999 [33]	13	II	A/Abo	200 U	Sweat quantification (DNSS) and VAS at 3 w, 8 w and 13 w	DNSS: Mean difference between A/Abo and PBO 34.5% (*p* < 0.001) at 3 w, 36.9% (*p* < 0.001) at 8 w, and 28.4% (*p* < 0.001) at 13 w. For VAS, 56.5% (*p* < 0.001) at 3 w, 67.4% (*p* < 0.001) at 8 w, and 62.5% (*p* < 0.001) at 13 w.	Pruritus in A/Abo axilla (2 pts) lasting for 1 w. Mild constipation and increased palmar sweating (2 pts for 1 w)
Heckmann *et al*., 2001 [35]	158	I	A/Abo	200 U	Reduction in sweat by gravimetry	Decrease in mean sweat production from 192 mg/min to 24 mg/min (*p* < 0.001).	No SAE
Naumann and Lowe 2001 [36]	320	I	A/Ona	50 U/axilla	Percentage of responders (spontaneous axillary sweat production >50% reduction from baseline at 4 w, patient GATS, and SAE	4 w—94% (227) of A/Ona group responded *vs.* 36% (28) of PBO. 16w—82% (198) A/Ona *vs.* 21% (16) in PBO. Higher patient satisfaction in A/Ona (*p*< 0.001). Adverse events: 27 (11%) in A/Ona, 4 (5%) in PBO (*p* > 0.05).	13% common cold and 5% compensatory sweating in A/Ona group.
Odderson 2002 [37]	18	II	A/Ona	100 U (50 U/ axilla)	Sweating per surface area (SPA) quantified monthly for 5 months.	A/Ona group showed average reduction in sweat production of 91.6% at 2 w (*p* < 0.05) and average reduction of 88.2% over 5 months	Mild compensatory hyperhidrosis between thighs in 1patient.
Baumann *et al*., 2005 [38]	23	III	B/Rima	2500 U (0.5 mL) per axilla	Safety, efficacy and duration of action using participant assessment of axillary hyperhidrosis improvement, QOL, physician assessment score at Day 30	Duration of action from 2.2–8.1 months (mean = 5 months). Day 30: Improvement in QOL score (*p* < 0.001) and physician assessment (*p* < 0.001).	Transient bruising, flu like symptoms, dry eyes, indigestion, menopausal bleeding in B/Rima group.
Lowe *et al*., 2007 [39]	322	II	A/Ona	75 U OR 50 U/axilla	HDSS	2-point improvement on the 4-point HDSS reported in 75% in the 75-U and 50-U A/Ona groups and in 25% of PBO (*p* < 0.001). Median duration of effect 197 days, 205 days, and 96 days in 75-U, 50-U, and PBO groups respectively.	No SAE reported

A/Ona: Onabotulinumtoxin A (Botox); A/Inco: incobotulinumtoxinA (Xeomin); A/Abo: abobotulinumtoxinA (Dysport); B/Rima: RimabotulinumtoxinB (Myobloc); DNSS: digitized ninhydrin-stained sheets; VAS: Visual Analogue Scale; SAE: Serious Adverse Effect; GATS: Patients Global assessments of treatment satisfaction; PBO: placebo; QOL: Quality of life; HDSS: Hyperhidrosis Disease Severity Scale; w: week(s).

### 7.1. Toxin *versus* Placebo (Table 3)

The first double-blind, placebo-controlled study of BoNT in hyperhydrosis was published in 1999 and encompassed 13 patients using A/Abo [34] with 50 U of toxin injected in each axilla. Sweat production was quantified by ninhydrin staining. Pain was measured by Visual Analogue Scale (VAS) after treatment. Significant sweat reduction was noted at 3, 8, and 13 weeks (*p* < 0.001). (Class II) 

Heckmann *et al*. [36] studied the effect of BoNT in 158 patients with AH using 200 U of A/Abo (100 in each axilla). Sweat reduction was quantified by gravimetry. Decrease in mean sweat production was significant (*p* < 0.001) at 2 and 24 weeks. No major adverse side effects were noted. (Class I).

In another study, Naumann *et al*. [37] investigated the effect of A/Ona in 320 patients by injecting 50 U into each axilla. This study was presented in two different papers that looked at different outcomes. The 2001 paper looked at percentage of responders (patients with >50% reduction from baseline spontaneous axillary sweat production) at 4 weeks, patients’ global assessment of treatment satisfaction score, and adverse events [37]. It was found that at 4 weeks, 94% (227) of the BoNT-A group had responded compared with 36% (28) of the placebo group. By week 16, response rates were 82% (198) and 21% (16), for toxin and placebo respectively. The results for all other measures of efficacy were significantly better in the BoNT-A group than the placebo group. Patient satisfaction was statistically significant at 4 weeks (*p* < 0.001). Adverse events were reported in 11% of BoNT-A group and encompassed 13% with common cold and 5% with compensatory sweating (*p* > 0.05). In the 2002 paper, outcome was measured via hyperhidrosis impact questionnaire, Medical Outcomes Trust Short Form-12 Health Survey (SF-12), objective percentage of responders and subjective global assessment of treatment satisfaction score [1]. Emotional status, ability to participate in daily and social activities, productivity at work and number of clothing changes per day showed statistical improvement (*p* < 0.01) at 1 week. This improvement was sustained with little or no decline throughout the 16-week visit. Improvement in the physical component summary score of the SF-12 was also statistically significant (*p* <0.019). (Class I) 

A small study of 18 patients [39] using 100 U (50 U in each axilla) of A/Ona also demonstrated significant sweat reduction (*p* < 0.05). The outcome was evaluated by measuring sweating per surface area quantified monthly for 5 months. By the end of the study only 1 out of 12 patients completing the study had returned to baseline sweat production. One patient did report mild compensatory hyperhidrosis between the thighs (Class II). 

Baumann *et al.* [17] studied effectiveness of B/Rima in 23 patients with AH using 2500 U of toxin per axilla. Safety, efficacy, and duration of action by participant assessment were evaluated as well as axillary hyperhidrosis improvement, quality of life score, and physician assessment score at day 30. Significant improvement in quality of life (*p* < 0.001) and improvement in physician assessment rating (*p* < 0.001) were noted at day 30. Side effects included bruising, flu like symptoms, dry eyes, indigestion and minor bleeds (two menopausal women) and were transient. Duration of action ranged from 2.2 to 8.1 months with a mean of 5 months. The placebo group was eliminated from the study due to the very small number of participants in this group (Class III). 

Lowe *et al.* [40] compared two different dosages of A/Ona (50 U *versus* 75 U/per axilla) with placebo in 322 patients with AH. The primary outcome was measured by Hyperhidrosis Disease Severity Scale (HDSS) and was monitored by telephone call or office visit at day seven, week four, week eight, and then every four weeks until the subject was eligible for reinjection or exited the study. Secondary outcome looked at gravimetric results and duration of effect. 2-point improvement on the 4-point HDSS was reported in 75% of subjects in the 75-U and 50-U BoNT-A groups and in 25% of the placebo group (*p* < 0.001). Median duration of effect was 197 days, 205 days, and 96 days in the 75-U, 50-U, and placebo groups, respectively. Of the subjects 78% completed the study. No major systemic side effects were reported (Class II).

### 7.2. Comparative Studies

#### 7.2.1. Toxin and Saline *versus* Toxin and Anesthetic

Seyedi and Simonart in 2007 performed a study of 29 patients by injecting 100 U of A/Ona reconstituted with saline in one axilla *versus* 100 U A/Ona mixed with lidocaine in the other [41]. Sweat was quantified by iodine-starch test and local pain from injection by VAS. Time of onset of sweat production, duration of effect and subjective percentage of mean decrease in sweating were similar for both groups. Pain score during the procedure was significantly lower in the axilla treated with lidocaine-reconstituted solution (*p* = 0.0027). Duration of anhidrosis in the axillae ranged from 4 to 7 months (mean 5.6). There was no difference in the duration of response between the two groups (*p* = 0.8125). No severe adverse events were noted in the study. Minor events included only hematoma at the site of injection and mild fatigue (Class II).

A preliminary result of another study comparing A/Ona mixed with saline against A/Ona mixed with lidocaine was published recently in 2012 encompassing eight patients with the focus on pain [42]. A total of 50 U of A/Ona were injected in each axilla. Primary outcomes included efficacy determined by gravimetric studies, safety as determined by adverse side effects, and pain determined by VAS score. Pain during the injections according to the VAS score was significantly lower in the axilla treated with lidocaine-diluted A/Ona. Amount of sweat reduction was similar in the lidocaine side *vs*. saline side by week 2 (18.5 ± 1.4 and 16.4 ± 1.2 respectively) and was statistically significant (*p* > 0.05). No side effects were recorded aside from occasional pain at the site of injection (Class II).

#### 7.2.2. Comparing Two Toxins

In a small study of 10 patients, Talarico-Filho injected 50 U of A/Ona into one axilla and 150 U of A/Abo into the other [43]. Minor’s test and gravimetry were performed at 0 days, 15 days, and monthly for 1 year for sweat assessment. Sweat rate was reduced by a mean of 97.7% for A/Ona and 99.4% for A/Abo. Duration of effect had a mean of 260 days for A/Ona and 290 days for A/Abo. Compensatory sweating did occur as a side effect although in minimal quantities, and not enough to interfere with quality of life (Class II). 

Dressler *et al.* [44] compared the effects of A/Ona to A/Inco toxins in 46 subjects after injecting 50 U of each in each axilla. Global self assessment scale, injection site pain, side to side difference in therapeutic effect and duration were evaluated. The overall therapeutic effect was reported as excellent in 89% of participants and good in another 11%. None of the patients reported minor or any side effects. The duration of the therapeutic effect was 3.2 ± 1.4 months and no differences were reported in a comparing side to side. All patients who started the study completed it; however, the end point was not specified. Patients were followed until onset of decrease in therapeutic effect (Class II). 

#### 7.2.3. Comparing Modes of Application

In a prospective, assessor blind study, Montaser-Kouhsari *et al.* [25] compared the effect of A/Abo injection with A/Abo iontophoresis in 11 patients with AH. A/Abo (250 U) was injected intradermally into one axilla and the same dose was iontophoresed in the other. The injection side had significantly less sweat production than the iontophoresis side at one week, one month, and six months after treatment (84%, 76%, and 50% *vs.* 73%, 22%, and 32%, respectively). Participants’ pain perception score during the procedure was significantly less on the iontophoresis side compared with the injection side (15.0 *vs.* 20.0, *p* < 0.05) (Class II).

### 7.3. Overall Level of Evidence in the Placebo Controlled and Comparator Studies

Based on these studies, the level of evidence is Level A (established) for BoNT-A collectively. However individually, level of evidence is Level B (probably effective) for A/Ona and A/Abo. For A/Inco the level is C (based on one class II comparator study) and for B/Rima the level is U as evidence is insufficient (only one class III study). (Table 4) 

**Table 4:** Axillary hyperhidrosis—overall level A for BoNT-A and level U for BoNT-B.

Toxin	Level of Evidence
A/Ona	Level B
A/Abo	Level B
A/Inco	Level C
B/Rima	Level U

## 8. Palmar Hyperhidrosis

Palmar hyperhidrosis (PH) is excessive sweating in the hands due to sympathetic cholinergic sudomotor nerve traffic to the palmar surface of the hands [15]. To date, seven blinded studies have evaluated the role of BoNTs in PH. Three studies compared injected toxin with placebo, two compared iontophoresis of A/Ona with placebo, one compared A/Ona with A/Abo (injection) and finally one compared two different doses of A/Ona. 

### 8.1. Toxin *versus* Placebo (Table 5)

Schnider *et al.* [12] studied A/Abo *versus* placebo in a small group of eleven patients. A total of 120 U was injected in one hand while the other hand received a comparative volume of placebo (saline). Primary outcome was evaluated through digitized ninhydrin sweat test and secondary outcome via VAS. Follow up lasted 13 weeks. Sweat production was found to decrease 26% at 3 weeks, 26% at 8 weeks, and 31% at 13 weeks (*p* < 0.001). Significant improvement on VAS was also noted. In the placebo arm, mean changes were not statistically significant and varied from 0.2% to 1.2%. Three patients reported transient and minor handgrip weakness. (Class II)

**Table 5:** Double blind palmar hyperhidrosis studies comparing toxin *vs.* placebo.

Author & Year	*N*	Class	Agent	Dose	Primary outcome	Result	Side effects
Schnider *et al*., 1997 [12]	11	II	A/Abo	120 U	DNSS	Mean reduction in sweat production: 26%, 26%, and 31% at w 3, 8, 13 (*p* < 0.001) and improvement in VAS 38%, 40%, and 35% at w 3, 8, 13 respectively (*p* = 0.002) for A/Abo group.	Minor , reversible weakness of handgrip lasting between 2 and 5 w, and minor hematoma at injection site
Lowe *et al*., 2002 [25]	19	II	A/Ona	100 U	Sweat production (gravimetric measurement) and physician’s and patient’s rating of severity. Safety evaluations via grip strength	Mean percentage change from baseline was significantly greater in the A/Ona-treated palms than in the PBO-treated palms at day 28 (*p* = 0.0037). Similar results found for Minor’s test.	Finger tingling and numbness in one A/Ona patient. One PBO patient with weakness of injected hand, one patient bilateral hand pain.
Baumann *et al*., 2005 [18]	20	II	B/Rima	5000 U	Efficacy, duration, safety, and patient and investigator assessment	Patient assessed efficacy showed significant difference in favor of B/Rima through day 120. Physician assessment did not show difference at day 30. Mean duration of effect; 3.8 months. Onset of effect: within 1 w.	Transient dry mouth in 18. Indigestion, dry hands. Muscle weakness in 12. Decreased grip strength in 10.

A/Ona: Onabotulinumtoxin A (Botox); A/Inco: incobotulinumtoxinA (Xeomin); A/Abo: abobotulinumtoxinA (Dysport); B/Rima: RimabotulinumtoxinB (Myobloc); DNSS: digitized ninhydrin-stained sheets; VAS: Visual Analogue Scale; SAE: Serious Adverse Effect; GATS: Patients Global assessments of treatment satisfaction; PBO: placebo; QOL: Quality of life; HDSS: Hyperhidrosis Disease Severity Scale; w: week(s).

In another study of nineteen subjects, Lowe *et al.* [45] injected 100 U A/Ona in one palm *versus* placebo in the other. Primary outcomes included change in sweat production (gravimetry), Minor’s iodine starch test and grip strength. Three patients were lost due to protocol violation but more than 80% of participants still completed the study. Reduction in sweat production was greater in the treatment arm (A/Ona) compared to placebo at day 28 (*p* = 0.0037). Similar results were found for Minor’s test as improvement in most patients occurred on day 7. One patient in the A/Ona group noted tingling and slight numbness in the hand, 1 reported mild hand weakness in the hand receiving placebo and 1 described pain in both hands. Patients were followed for 28 days (Class II).

In 2005, Baumann *et al.* [46] investigated the effect of 5000 U of B/Rima *versus* placebo in 20 patients with PH. Patient and physician assessed efficacy and duration of effect up to 120 days. Patient assessed change in efficacy showed significant difference in favor of B/Rima at different time points lasting to day 120. Onset of effect was observed at week 1. Mean duration of efficacy was 3.8 months. Systemic side effects were common (dry mouth in 18 patients) and included indigestion. Twelve patients reported muscle weakness and 10 had decreased hand grip (Class II).

In a double blind study of 8 patients, Kavanaugh and Shams [26] iontophoresed 100 U of A/Ona (diluted in 2.7 mL of preservative free saline) into the patient’s dominant hand and the same volume of saline into the non-dominant hand. At 14 days, the sweat rate was significantly reduced (34%) (*p* < 0.05) in the hands with toxin exposure whereas the saline hand showed subtle increase in sweating (Class II). Davarian *et al*. [24] also conducted a double blind study in eight patients with severe palmar hyperhidrosis using iontophoresis, administering A/Abo into the dominant hand and normal saline in the other hand. Gravimetry and iodine-starch tests were performed to evaluate the rate of sweating. The evaluation sessions were at baseline, 2 and 4 days, 1, 2, 3, 4, 8 and 12 weeks after treatment. The patients were also asked to fill out the Dermatology Life Quality Index questionnaire at 2, 4, 8 and 12 weeks after treatment. They reported marked reduction of sweating rate at the first, second, third and fourth week, as well as at the second and third month for the toxin treated hand (*p* < 0.05) when compared to the 3rd and 4th week for saline treated hand (*p* < 0.05). Overall, the treated (right) hand showed a greater reduction than the placebo (left) hand at all sessions but was only found to be statistically significant at week 12 after treatment (*p* = 0.03). No hand weakness or other side effect was noted (Class II).

### 8.2. Comparative Studies

Simonetta and Moreau [47] compared A/Ona injection in one palms *versus* A/Abo injection in the other palm in 8 patients using a 1:4 dosing ratio. The mean dose of A/Ona per palm was 69 and the mean dose of A/Abo was 284 U per palm. Follow-up was for 6 months and no patients were lost to follow-up. Outcomes measured quantification of sweat production by Minor's iodine starch sweat test at 1, 3, and 6 months after injection and subjective assessment by VAS. Patients demonstrated a mean palmar sweating area (PSA) of 76.8% for A/Abo (*p* = 0.002) *versus* 56.6% for A/Ona (*p* = 0.003) at 1 month. At 3 mo, decrease in PSA was 69.4% for A/Abo (*p* = 0.008) and 48.8% for A/Ona (NS). Mean duration of benefit was 17 weeks for A/Abo; 18 weeks for A/Ona. Weakness of finger-pincher index occurred in both groups (4 in A/Abo and 2 in A/Ona group). The study lasted 6 months. Investigators used ulnar and median nerve blocks to decrease the pain of injection (Class II).

In another study by Saadia *et al*. [15], the effect of two doses of A/Ona, 50 and 100 units was compared with each other. Primary outcome was measured by Iodine starch test at 1, 3, and 6 months. Handgrip and finger pinch strength were also evaluated. Patients were followed for 6 months. A significant decrease in sweating was found within a month. Anhidrotic effect was significant up to 6 months in the 50 U group and up to 5 months in the 100 U group. Treatment lasted between 2 and 6 months. Investigators were not blinded to injections but did not participate in statistical analysis. Efficacy was similar for both doses. Objective assessment of grip strength showed no significant weakness in patients of either dose group. Thumb-index pinch strength decreased in both groups but also improved gradually by six months (Class II).

### 8.3. Overall Level of Evidence in Placebo-Controlled and Comparator Studies

Prospective and blinded studies with BoNTs in palmar hyprehidrosis are limited. In injection studies, there are three class II studies for A/Ona , two class II for A/Abo and one class II for B/Rima. In iontophoretic studies, there is one class II study for each A/Ona and A/Abo toxins. Collectively, the studies depict a level B evidence (probably effective) for BoNT-A and level C evidence (possibly effective) for BoNT-B in PH (Table 6).

**Table 6:** Palmar hyperhidrosis—overall level B for BoNT-A and level C for BoNT-B.

Toxin	Level of Evidence
A/Ona	Level B
A/Abo	Level B
A/Inco	Level U
B/Rima	Level C

## 9. Gustatory Hyperhidrosis

Gustatory sweating is a common post-surgical complaint occurring in patients after parotidectomy usually for adenoma. It presents as a component of Frey’s syndrome, which also includes parotid flushing. Prevalence is as much as 100% post parotidectomy as reported in the literature [48]. This phenomenon may be secondary to misdirected regeneration of postganglionic nerve fibers of both the auriculotemporal and greater auricular nerves. Once the nerve is injured, nerve fibers connect to injured postganglionic sympathetic nerve fibers ultimately innervating sweat glands and small skin vessels and creating a misdirected reflex action portrayed as profuse sweating after gustatory stimulation [49]. Gustatory sweating has an adverse effect on quality of life due to its inevitable appearance in affected patients. Systemic or topical anticholinergics and topical application of aluminum salts may provide some relief [49]. Surgical options include cervical sympathectomy, resection of the tympanic plexus, and resection of the glossopharyngeal nerve [50]. 

Retrospective studies depict marked and long-lasting improvements in facial sweating ranging from 11 to 36 months after a single injection of A/Ona in 78 subjects studied [49,51]. Discrepancies still exist over exact dosage to use and exactly how long the duration of the effect is. 

## 10. Compensatory Hyperhidrosis

Compensatory hyperhidrosis can be an adverse effect of thoracic sympathectomy and can be equally as debilitating as idiopathic hyperhidrosis. Two recent case reports describe the use of low doses of BoNT-A to treat compensatory hyperhidrosis following thoracic sympathectomy with high level of satisfaction and abolishment of sweating for up to 10 months [52]. 

## 11. Surgical Therapy

Surgical approaches range from local excision of the gland to sympathectomy. Local excision of the gland or subcutaneous curettage is performed for axillary hyperhidrosis and can be done under local anesthesia. Long-term follow-up in a large number of patients is not available [53]. The established approach, especially for palmar hyperhidrosis, is endoscopic transthoracic sympathectomy (ETS) with resection at T2 and T3 levels commonly used [54]. One double-blind, randomized controlled trial compared the effectiveness of resecting T2-T3 *versus* T2 alone and found that T2 ablation is equally as effective as T2-T3 ablation in terms of symptomatic relief, recurrence, compensatory hyperhidrosis, and patient satisfaction [38]. Although a variant of this procedure can be performed for plantar hyperhidrosis where L3 is removed, high risk of sexual dysfunction makes this option less appealing [55]. Compensatory hyperhidrosis, Horner’s syndrome, gustatory sweating, neuralgia, and pneumothroax are all possible side effects of ETS [37]. 

## 12. Conclusion and Clinical Comments

Topical and oral agents are probably effective (Level B) in hyperhidrosis and often are tried as first line remedies. Clinical practice and data from blinded studies indicate an efficacy level of B (probably effective) for A/Ona and A/Abo, Level C (possibly effective) for A/Inco and insufficient evidence (level U) for B/Rima in AH. The effective dose for A/Ona, A/Inco and A/Abo is in the range of 50–75, 100–200 and 50–75 units per axilla respectively. The effect after one injection can last 3–9 months. Most BoNT practitioners find BoNT injections also fairly effective for PH. The limited number of blinded studies in the literature combining BoNT use via injection and iontophoresis in PH indicate level B evidence (probably effective) for A/Ona and A/Abo, insufficient evidence for A/Inco and level C evidence (possibly effective) for B/Rima. More systemic side effects are reported with B/Rima injections, an issue that deserves further investigation. 

In practice, most adults with AH or PH endure the pain of injections and find the benefit out weighing the discomfort. In teenagers (who constitute a sizeable number of patients with primary hyperhydrosis) however, pain is often not acceptable and the return rate for treatment is low. The new data with iontophoresis [24,25,26] are encouraging and may particularly prove useful for young individuals with this condition. Unfortunately, the magnitude of the response with iontophoresis is still suboptimal and less than that seen with the injection technique (only 30%–35% sweat reduction beyond two weeks). Refinement of the iontophoresis technique may lengthen the duration of response in AH and PH and prove to be especially helpful in young patients. 

## 13. A Technical Note from the Senior Author

At Yale New Haven Hospital botulinum toxin treatment clinic, we use 75–100 units of A/Ona per palm or axilla. Our scheme of injection includes two additional rows when compared to that seen in Figure 1 (each row 4–5 injection points) at the mid-palm. Patients prepare their skin with Emla cream two hours before injection and we use anesthetic spray for two to three rows at a time just before each injection. The satisfaction rate with adults has been over 90%. 

## References

[1] Naumann M.K., Hamm H., Lowe N.J. (2002). Botox Hyperhidrosis Clinical Study Group. Effect of botulinum toxin type a on quality of life measures in patients with excessive axillary sweating: A randomized controlled trial. Br. J. Dermatol..

[2] Leung A.K.C., Chan P.Y.H., Choi M.C.K. (1999). Hyperhidrosis. Int. J. Dermatol..

[3] Adar R. (1977). Palmar hyperhidrosis and its surgical treatment: A report of 100 cases. Ann. Surg..

[4] Del Sorbo F., Brancati F., de Joanna G., Valente E.M., Lauria G., Albanese A. (2011). Primary focal hyperhidrosis in a new family not linked to known loci. Dermatology.

[5] Hornberger J., Grimes K., Naumann M., Glaser D.A., Lowe N.J., Naver H., Ahn S., Stolman L.P. (2004). Recognition, diagnosis, and treatment of primary focal hyperhidrosis. J. Am. Acad. Dermatol..

[6] Bachmann M., Myers J.E., Bezuidenhout B.N. (1992). Acrylamide monomer and peripheral neuropathy in chemical workers. Am. J. Ind. Med..

[7] Sato K., Kang W.H., Saga K., Sato K.T. (1989). Biology of sweat glands and their disorders. I. Normal sweat gland function. J. Am. Acad. Dermatol..

[8] Strutton D.R., Kowalski J.W., Glaser D.A., Stang P.E. (2004). Us prevalence of hyperhidrosis and impact on individuals with axillary hyperhidrosis: Results from a national survey. J. Am. Acad. Dermatol..

[9] French J., Gronseth G. (2008). Lost in a jungle of evidence: We need a compass. Neurology.

[10] Sato K., Kang W.H., Saga K., Sato K.T. (1989). Biology of sweat glands and their disorders. II. Disorders of sweat gland function. J. Am. Acad. Dermatol..

[11] Walling H.W. (2009). Primary hyperhidrosis increases the risk of cutaneous infection: A case-control study of 387 patients. J. Am. Acad. Dermatol..

[12] Schnider P., Binder M., Auff E., Kittler H., Berger T., Wolff K. (1997). Double-blind trial of botulinum a toxin for the treatment of focal hyperhidrosis of the palms. Br. J. Dermatol..

[13] Sato K., Sato F. (1987). Effect of vip on sweat secretion and camp accumulation in isolated simian eccrine glands. Am. J. Physiol..

[14] Rothman S. (1954). Physiology and Biochemistry of the Skin.

[15] Saadia D., Voustianiouk A., Wang A.K., Kaufmann H. (2001). Botulinum toxin type a in primary palmar hyperhidrosis: Randomized, single-blind, two-dose study. Neurology.

[16] Shelley W.B., Hurley H.J. (1975). Studies on topical antiperspirant control of axillary hyperhidrosis. Acta Derm. Venereol..

[17] Baumann L., Slezinger A., Halem M., Vujevich J., Mallin K., Charles C., Martin L.K., Black L., Bryde J. (2005). Double-blind, randomized, placebo-controlled pilot study of the safety and efficacy of myobloc (botulinum toxin type b) for the treatment of palmar hyperhidrosis. Dermatol. Surg..

[18] Try C., Messikh R., Elkhyat A., Aubin F., Humbert R.P. (2012). Use of oral oxybutynin at 7.5 mg per day in primary hyperhidrosis. Rev. Méd. Liege.

[19] Walling H.W., Swick B.L. (2011). Treatment options for hyperhidrosis. Am. J. Clin. Dermatol..

[20] Ghaleiha A., Jahangard L., Sherafat Z., Ahmadpanah M., Brand S., Holsboer-Trachsler E., Bajoghli H., Haghighi M. (2012). Oxybutynin reduces sweating in depressed patients treated with sertraline: A double-blind, placebo-controlled, clinical study. Neuropsychiatr. Dis. Treat..

[21] Hund M., Sinkgraven R., Rzany B. (2004). Randomized, placebo-controlled, double blind clinical trial for the evaluation of the efficacy and safety of oral methantheliniumbromide (vagantin) in the treatment of focal hyperhidrosis. J. Ger. Soc. Dermatol..

[22] Stolman L.P. (1987). Treatment of excess sweating of the palms by iontophoresis. Arch. Dermatol..

[23] Stolman L.P. (2008). Hyperhidrosis: Medical and surgical treatment. Eplasty.

[24] Davarian S., Kalantari K.K., Rezasoltani A., Rahimi A. (2008). Effect and persistency of botulinum toxin iontophoresis in the treatment of palmar hyperhidrosis. Australas. J. Dermatol..

[25] Montaser-Kouhsari L., Zartab H., Fanian F., Noorian N., Sadr B., Nassiri-Kashani M.,  Firooz A. (2013). Comparison of intradermal injection with iontophoresis of abobotulinum toxin a for the treatment of primary axillary hyperhidrosis: A randomized, controlled trial. J. Dermatolog. Treat..

[26] Kavanagh G.M., Shams K. (2006). Botulinum toxin type a by iontophoresis for primary palmar hyperhidrosis. J. Am. Acad. Dermatol..

[27] Freeman R., Waldorf H.A., Dover J.S. (1992). Autonomic neurodermatology (part ii): Disorders of sweating and flushing. Semin. Neurol..

[28] Reinauer S., Neusser A., Schauf G., Holzle E. (1993). Iontophoresis with alternating current and direct current offset (ac/dc iontophoresis): A new approach for the treatment of hyperhidrosis. Br. J. Dermatol..

[29] Holzle E. (2012). Tap water iontophoresis. Hautarzt Z. Dermatol. Venerol. Verwandte Geb..

[30] Bushara K.O., Park D.M., Jones J.C., Schutta H.S. (1996). Botulinum toxin—A possible new treatment for axillary hyperhidrosis. Clin. Exp. Dermatol..

[31] Jankovic J. (2004). Treatment of cervical dystonia with botulinum toxin. Move. Disord..

[32] Mannava S., Mannava K.A., Nazir O.F., Plate J.F., Smith B.P., Koman L.A., Tuohy C.J. (2013). Treatment of palmar hyperhidrosis with botulinum neurotoxin a. J. Hand Surg..

[33] Lim D., Bekhor P., Webber S., Manohara S., Rodrigues M., Cartmill A. BTX Injections for Excessive Sweating. http://www.sweatfree.com.au/sweat-treatments/botox-underarm-sweating2012.

[34] Schnider P., Binder M., Kittler H., Birner P., Starkel D., Wolff K., Auff E. (1999). A randomized, double-blind, placebo-controlled trial of botulinum a toxin for severe axillary hyperhidrosis. Br. J. Dermatol..

[35] Champion R.H. (1986). Disorders of sweat glands. Textb. Dermatol..

[36] Heckmann M., Ceballos-Baumann A.O., Plewig G. (2001). Botulinum toxin a for axillary hyperhidrosis (excessive sweating). N. Engl. J. Med..

[37] Naumann M., Lowe N.J. (2001). Botulinum toxin type a in treatment of bilateral primary axillary hyperhidrosis: Randomised, parallel group, double blind, placebo controlled trial. BMJ.

[38] Katara A.N., Domino J.P., Cheah W.K., So J.B., Ning C., Lomanto D. (2007). Comparing t2 and t2-t3 ablation in thoracoscopic sympathectomy for palmar hyperhidrosis: A randomized control trial. Surg. Endosc..

[39] Odderson I.R. (2002). Long-term quantitative benefits of botulinum toxin type a in the treatment of axillary hyperhidrosis. Dermatol. Surg..

[40] Lowe N.J., Glaser D.A., Eadie N., Daggett S., Kowalski J.W., Lai P.Y. (2007). Botulinum toxin type a in the treatment of primary axillary hyperhidrosis: A 52-week multicenter double-blind, randomized, placebo-controlled study of efficacy and safety. J. Am. Acad. Dermatol..

[41] Vadoud-Seyedi J., Simonart T. (2007). Treatment of axillary hyperhidrosis with botulinum toxin type a reconstituted in lidocaine or in normal saline: A randomized, side-by-side, double-blind study. Br. J. Dermatol..

[42] Gulec A.T. (2012). Dilution of botulinum toxin a in lidocaine *vs*. in normal saline for the treatment of primary axillary hyperhidrosis: A double-blind, randomized, comparative preliminary study. J. Eur. Acad. Dermatol. Venereol..

[43] Talarico-Filho S., Mendonça DO Nascimento M., Sperandeo DE Macedo F., De Sanctis Pecora C. (2007). A double-blind, randomized, comparative study of two type a botulinum toxins in the treatment of primary axillary hyperhidrosis. Dermatol. Surg..

[44] Dressler D. (2010). Comparing botox and xeomin for axillar hyperhidrosis. J. Neural Transm..

[45] Lowe N.J., Yamauchi P.S., Lask G.P., Patnaik R., Iyer S. (2002). Efficacy and safety of botulinum toxin type a in the treatment of palmar hyperhidrosis: A double-blind, randomized, placebo-controlled study. Dermatolog. Surg..

[46] Baumann L., Slezinger A., Halem M., Vujevich J., Martin L.K., Black L., Bryde J. (2005). Pilot study of the safety and efficacy of myobloc (botulinum toxin type b) for treatment of axillary hyperhidrosis. Int. J. Dermatol..

[47] Simonetta Moreau M., Cauhepe C., Magues J.P., Senard J.M. (2003). A double-blind, randomized, comparative study of dysport *vs*. Botox in primary palmar hyperhidrosis. Br. J. Dermatol..

[48] Laage-Hellman J.E. (1957). Gustatory sweating and flushing after conservative parotidectomy. Acta Oto Laryngol..

[49] Eckardt A., Kuettner C. (2003). Treatment of gustatory sweating (frey’s syndrome) with botulinum toxin a. Head Neck.

[50] Haxton H.A. (1948). Gustatory sweating. Brain.

[51] Naumann M., Zellner M., Toyka K.V., Reiners K. (1997). Treatment of gustatory sweating with botulinum toxin. Ann. Neurol..

[52] Santana-Rodriguez N., Clavo-Varas B., Ponce-Gonzalez M.A., Jarabo-Sarceda J.R., Perez-Alonso D., Ruiz-Caballero J.A., Olmo-Quintana V., Atallah Yordi N., Fiuza-Perez M.D. (2012). Primary frontal hyperhidrosis successfully treated with low doses of botulinum toxin a as a useful alternative to surgical treatment. J. Dermatolog. Treat..

[53] Furlan A.D., Mailis A., Papagapiou M. (2000). Are we paying a high price for surgical sympathectomy? A systematic literature review of late complications. J. Pain.

[54] Riolo J., Gumucio C.A., Young A.E., Young V.L. (1990). Surgical management of palmar hyperhidrosis. South. Med. J..

[55] Stolman L.P. (1998). Treatment of hyperhidrosis. Dermatol. Clin..

